# Influence of high cyclic ambient temperature and water drinker design on growth performance and water disappearance of growing-finishing pigs

**DOI:** 10.1093/tas/txac117

**Published:** 2022-08-24

**Authors:** Katherine D Vande Pol, Nicholas S Grohmann, Thomas E Weber, Matthew J Ritter, Michael Ellis

**Affiliations:** Department of Animal Science, University of Illinois, Urbana-Champaign, IL 61801, USA; Department of Animal Science, University of Illinois, Urbana-Champaign, IL 61801, USA; Elanco Animal Health, Greenfield, IN 46140, USA; Elanco Animal Health, Greenfield, IN 46140, USA; Department of Animal Science, University of Illinois, Urbana-Champaign, IL 61801, USA

**Keywords:** ambient temperature, drinker design, growing-finishing pigs, growth, water disappearance

## Abstract

The objective of this study was to determine effects of room temperature and drinker design on growth and water disappearance of growing-finishing pigs (26.9 ± 3.67 to 130.9 ± 5.10 kg live body weight). A split-plot design was used with a 2 × 2 factorial arrangement of treatments: Room Temperature (RT) [Thermoneutral (TN) vs. High (HI); main plot], Drinker Design (DD; Nipple vs. Cup; sub-plot). A total of 316 pigs were used, housed in 32 pens in 4 rooms (8 pens/room; 7 pens of 10 pigs and 1 pen of 9 pigs). Two rooms were on each RT treatment. Room temperature for the TN treatment was constant throughout each day but decreased from 24°C at the start to 20°C and 18°C on d 14 and 45 of the study period, respectively. For the HI treatment, a single, cyclic RT protocol was used throughout the study (30°C from 08:00 to 19:00 h and 20°C from 20:00 to 07:00 h, with 1-h transition periods). Pens had fully-slatted concrete floors and 1 feeder and drinker (either nipple or cup); floor space was 0.67 m^2^/pig. Pigs had ad libitum access to standard corn-soybean diets, formulated to meet or exceed NRC (2012) nutrient requirements. Water disappearance was measured using a meter fitted to the water line supplying each drinker. There were no interactions (*P* > 0.05) between RT and DD treatments. Drinker Design did not affect (*P* > 0.05) growth performance; water disappearance was 7.3% greater (*P* ≤ 0.05) for Nipple than Cup drinkers. Compared to the TN treatment, the HI treatment had no effect (*P* > 0.05) on gain:feed ratio, but resulted in lower (*P* ≤ 0.05) average daily gain (6.5%) and average daily feed intake (5.5%) and greater (*P* ≤ 0.05) average daily water disappearance (16.8%). These results suggest that both drinker design and RT can affect water disappearance, and that the high, cyclic RT regime used reduced growth performance of growing-finishing pigs. Further research is needed to determine the contribution of water intake and wastage to treatment differences in water disappearance.

## INTRODUCTION

High environmental temperatures are commonly experienced in many areas of the world where pigs are produced, particularly in the summer months, and these temperatures negatively impact the performance of growing-finishing pigs (Renaudeau et al., 2011). It has been suggested that the impact of hot conditions on the performance of pigs is likely to become of greater importance in the future due to a combination of climate change and the development of pig production in tropical and sub-tropical areas of the world (Campos et al., 2017). Although there has been a significant amount of research carried out to establish the impact of high temperatures on the performance of growing-finishing pigs, most studies evaluated the effect of high constant temperatures. However, in commercial practice, pigs generally experience diurnal fluctuations in ambient temperature under hot conditions. Renaudeau et al. (2011) reported that only 16% of studies investigating the effects of high ambient temperature on growth performance that were published between 1960 and 2009 evaluated cyclic temperature regimes. Also, many of these studies were carried out using individually-housed pigs and under conditions that are considerably different from those on commercial facilities (Le Bellego et al., 2002). In addition, there has been substantial change in the genetic potential of pigs over recent decades. There is evidence that genetic lines with high compared to moderate lean growth potential have higher levels of heat production (Nienaber et al., 1997) which would influence the responses of growing-finishing pigs to high environmental temperatures.

As well as influencing growth, high ambient temperatures can also impact water intake (Lopez et al., 1991), but there are relatively few published studies quantifying the relationship between these two factors. In addition, there has been limited recent research evaluating other major factors that can influence water intake such as drinker design. Quantifying the relationship between environmental factors and water intake is important for producers. Water intake levels affect the delivery of medication and nutrients via the water supply (Little et al., 2019). Estimates of intake levels are also needed to determine facility-level water usage to ensure systems are designed and operated to supply sufficient water to the animals. In addition, water disappearance may be an early indicator of animal health status (Pedersen and Madsen, 2001), and an understanding of typical water disappearance levels at different environmental temperatures and for commonly used drinker designs are needed to allow producers to identify atypical situations. The objective of this study was to evaluate the impact of cyclic high environmental temperature and of drinker design on the growth performance and water disappearance of growing-finishing pigs.

## MATERIALS AND METHODS

This study was conducted in a wean-to-finish building at the swine research facilities of the University of Illinois. The experimental protocol was approved by the University of Illinois Institutional Animal Care and Use Committee prior to the initiation of the research.

### Experimental Design, Treatments, and Treatment Allocation

The study was carried out from the start of wk 8 post-weaning (26.9 ± 3.67 kg body weight) to the end of wk 24 post-weaning (130.9 ± 5.10 kg body weight) using a total of 316 pigs. A split-plot design was used with a 2 × 2 factorial arrangement of treatments: Room Temperature [RT; main plot; Thermoneutral (TN) vs. High (HI)]; and Drinker Design (DD; sub-plot; Nipple vs. Cup). For the TN treatment, a constant temperature regime was used over the 24 h period. At the start of the study, the thermostat controlling the room temperature for this treatment was set at 24°C. This setting was reduced to 20 and 18°C on the day after the pigs reached an average body weight (BW) of 35 and 70 kg, respectively, which was on d 14 and 45 of the study, respectively. The thermostat setting remained at 18°C from d 45 to the end of the study (end of wk 24 post-weaning). For the HI treatment, the same diurnal temperature regime were used throughout the study period. The thermostat was set at 30°C from 08:00 to 19:00 h and at 20°C from 20:00 to 07:00 for each day. There was a 1-h transition period at both 19:00 and 07:00 h, during which the thermostat setting adjusted gradually between the two temperatures.

Four identical rooms with 8 pens in each room were used. Seven of the pens in each room housed 10 pigs. However, one pen in each room was smaller than the others; consequently, only 9 pigs were housed in those pens to maintain a similar floor space per pig across all pens. Prior to the allotment of the pigs to the study, the rooms were randomly allocated to RT treatment and 2 adjacent pens within each room were randomly allocated to the DD treatments. Pigs were allotted to groups at the start of wk 7 post-weaning with replicates consisting of 4 groups, 1 group of each DD within each RT, resulting in 2 replicates for the main plot of RT and 16 replicates for the sub-plot of DD. Prior to allotment, pigs were housed in pens of 21. Two pens with a similar mean and CV of BW were selected, and each pen of pigs was split into 2 groups of 10 pigs each (with the extra pig being removed) such that the 4 groups within a replicate contained 10 pigs with a similar mean and CV of BW, and the same ratio of barrows and gilts. Within a replicate, two groups were randomly allotted to each of the RT treatments, and 1 of these 2 groups was randomly allotted to a DD treatment pen. For the pens that housed 9 pigs, the one pig closest in BW to the pen mean was removed such that the pen average and CV of BW was similar to the other pens in the replicate. Pigs were allowed a 1-wk acclimation period between allotment and the start of the study to adjust to the new conditions. The DD treatments were applied at allotment; the RT treatments were not applied until the start of the study period which was at the beginning of wk 8 post-weaning.

### Animals, Housing, and Management

Pigs used in the study were the progeny of Genetiporc Fertilis 25 sows mated to G-Performer Line sires (PIC; Hendersonville, TN, USA). Pens had fully-slatted concrete floors; the pen divisions were constructed of vertical metal bars. Pen dimensions were 1.83 m by 3.66 m and 1.60 m by 3.66 m for pens housing 10 and 9 pigs, respectively, giving a total floor space of 6.70 and 5.86 m^2^, respectively, and a floor space per pig of 0.67 and 0.65 m^2^, respectively. Each pen was equipped with a 2-hole dry box feeder (Farmweld, Teutopolis, IL, USA) and, depending on DD treatment, either one nipple-type water drinker (Edstrom Hog Nipple; Avidity Science, Waterford, WI, USA) or one cup-type water drinker (Farmweld DRIK-O-MAT Wean-to-Finish Cup; Farmweld, Teutopolis, IL, USA).

The height of the cup drinkers was not adjustable and was set at the manufacturer’s recommendation for wean-to-finish pigs (102 mm from the floor to the bottom lip of the cup). The height for the nipple drinkers (distance from floor to lowest point of nipple drinker) was set at the start of the study at 50 mm above the shoulder height of the lightest pig in the pen, based on the study of Li et al. (2005). Shoulder height of the lightest pig in each pen was calculated according to the equation of Petherick (1983): shoulder height (mm) = 150 × (BW in kg)^0.33^. Nipple drinker heights were adjusted every 2 wk during the study based on the most recent pig weights.

Ambient temperature in each room was maintained using heaters, evaporative cooling cells, and fan ventilation as needed. Thermostats for each room were set according to RT treatment. The HI treatment temperatures were selected to be typical of temperatures experienced in the Midwest region of the United States during the summer months. Historic weather data suggested that the average daily high temperature in Illinois during the month of July was 29.6 °C and average nighttime temperature was 18.3°C (Illinois Climate Network, 2021). The TN treatment temperatures were based on the recommendations of the Guide for the Care and Use of Agricultural Animals in Research and Teaching (2010). Environmental temperature and humidity in each room were recorded twice daily throughout the study period using HOBO H8 data loggers (Onset, Bourne, MA, USA).

With the exception of the RT treatments, management during the study period was according to standard unit protocols, which were in line with commercial practices. The facility was illuminated via ambient and artificial lighting. Security lighting was provided 24 h each day, with additional lighting available when the investigators were working with the animals. Natural light via windows was present during daylight hours. Pigs had ad libitum access to standard corn-soybean meal-based diets in meal form that were formulated to meet or exceed NRC (2012) recommendations for nutrient requirements. Pigs had ad libitum access to water throughout the study period. Feeders and drinkers were checked daily for proper function and cleaned as necessary. Pigs were checked twice daily, and any pig requiring intervention was treated in accordance with the recommendations of the attending veterinarian. Any pig that was removed from the study was weighed, and the date of removal was recorded; this information was included in the calculation of average daily gain, average daily feed intake, and gain:feed ratio.

### Measurements

Average daily water disappearance (ADWD) was measured daily throughout the study utilizing water flow meters (Assured Automation ½ inch water meter, model WMP-P-050; Assured Automation, Roselle, NJ, USA). One meter was installed in the pipeline supplying water to the drinker in each pen; meter readings were recorded daily at approximately 07:00 h. All water meters were validated for accuracy at the start of and every 2 wk during the study period. For this validation, a total of 22.7 L of water was collected: 3.8 L directly from the drinker and 18.9 L from the water line between the meter and the drinker. Meter readings were taken at the start and end of the collection and the difference between these readings was compared to the measured quantity of water collected. If the error (i.e., the difference between the meter readings and the amount of water collected) was greater than 5%, the meter validation procedure was repeated. If the error continued to exceed 5%, the meter was replaced, and the validation procedure was carried out on the replacement meter.

The water flow rates to the drinkers were set at 1 L/min for both DDs which is a level generally recommended for growing-finishing pigs (Patience, 2012). Flow rates were checked at the start of and every 2 wk during the study by collecting and measuring the amount of water delivered from each drinker over a 1 min period; flow rates were adjusted if needed. Over the study period, flow rates averaged 1.01 ± 0.143 L/min for the cup drinkers and 1.07 ± 0.156 L/min for the nipple drinkers.

Pigs were individually weighed using a digital scale (Digi-Star model SW4600EID scale; Digi-Star LLC, Fort Atkinson, WI, U.S.A.; accurate to 0.2 kg) at the start of the study (start of wk 8 post-weaning) and every 2 wk during the study period until the average BW of the pigs in the pen reached 127 ± 2.0 kg. At this time, the two heaviest pigs were removed from the pen. This approach is in line with commercial practice where it is common to remove the heaviest pigs from a group for harvest prior to the end of the finishing period. The remaining pigs in the pen continued on the study and were weighed weekly until the end of wk 24 post-weaning, at which point all pigs in the pen were taken off-test. All scales used for measurement of pig weight were validated for accuracy using certified check weights that approximated to the expected weight of the pigs at the time of weighing. All additions of feed to the feeders were recorded and feeders were weighed each time pig weights were collected to calculate pen feed intake.

Respiration rates were measured to verify that the temperatures used for the HI treatment exceeded the thermal comfort zone of the pigs and caused heat stress. Measurements were recorded once per wk at approximately 12:00 h on a randomly-selected sub-sample of 2 pigs from 4 randomly-selected pens from each RT treatment. Respiration rate was measured on pigs that were in the recumbent position using a stopwatch and counting the flank movements for a 30 s period; this number was multiplied by 2 to give breaths/min.

### Statistical Analysis

The pen of pigs was the experimental unit for all measurements. The PROC UNIVARIATE procedure of SAS (SAS Inst. Inc., Cary, NC) was used to verify normality and homogeneity of variances of the residuals. All variables conformed to the assumptions of normality and homogeneity and were analyzed using the PROC MIXED procedure of SAS (Littell et al., 1996). The study was carried out using a split-plot design; the model accounted for the fixed effects of RT (main plot), DD (sub-plot), and the interaction, and the random effects of replicate and all two- and three-way interactions with replicate. Room was the experimental unit for RT; pen was the experimental unit for DD. The error term to test the effect of RT was the RT by replicate interaction; the error term to test the effect of DD and the RT by DD interaction was the sum of the DD by replicate interaction and RT by DD by replicate interaction. Differences between least-squares means were separated using the PDIFF option of SAS and were considered significant at *P* ≤ 0.05. *P*-values were adjusted using a Tukey’s adjustment for multiple comparisons.

In addition, regression analyses were conducted to determine relationships between respiration rate and day of study, between average daily gain (ADG), average daily feed intake (ADFI), gain:feed ratio (G:F), and ADWD and BW, and between ADWD and ADFI for the two RT. The PROC REG procedure of SAS was used; models used for the dependent variables (i.e., respiration rate, ADG, ADFI, G:F, or ADWD) included RT treatment, the independent variable (i.e., day of study, BW, or ADFI), and the interaction of RT and the independent variable. Independent variables were included as first-, second-, and third-order terms, with the higher-order terms being removed from the model if *P* > 0.05. For all analyses, the regression coefficients for the TN treatment were estimated first, and then the adjustments to these coefficients were determined for the HI treatment. Coefficients and adjustments were considered different to zero at *P* ≤ 0.05; adjustments indicated differences between RT treatments for the respective term.

## RESULTS AND DISCUSSION

**Figure 1.**
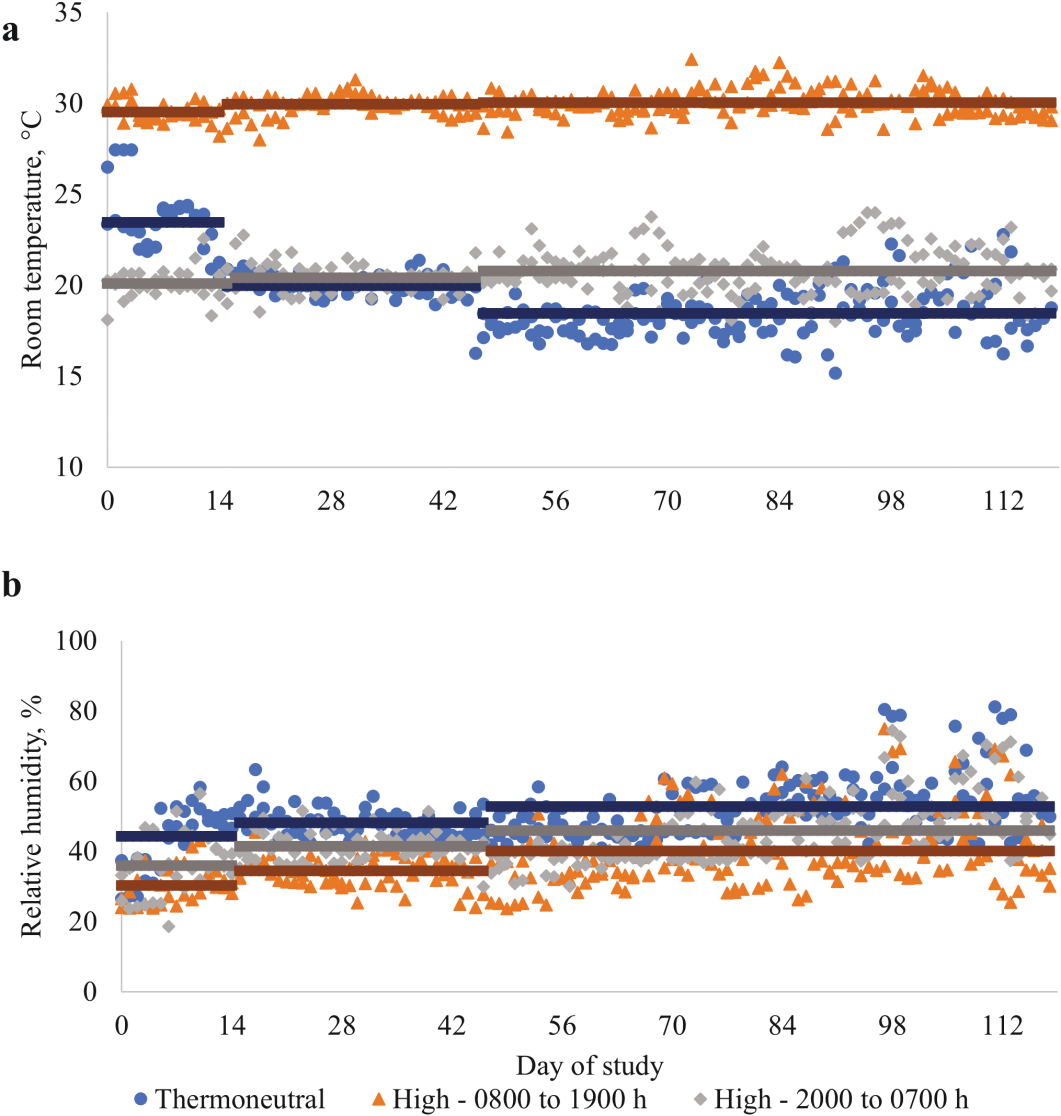
Ambient temperature and relative humidity for the Room Temperature treatment over the study period (8 to 25 wk post-weaning). **(a)** Average daily RT. (**b**) Average daily relative humidity. Horizontal lines indicate the mean temperature or relative humidity for each study period. Thermoneutral = thermostat set at constant temperature according to pig body weight (24 °C from the start to 35 kg live BW, 20 °C from 35 to 70 kg live BW, and at 18 °C for the remainder of the study period); High = throughout the study period the thermostat was set at 30 °C from 08:00 to 19:00 h and at 20 °C from 20:00 to 07:00, with 1-h transition periods.

Daily temperature and relative humidity levels during the study period for each of the RT treatments, averaged across the two rooms, are presented in Table 1 and Figure 1. The average daily RT for the overall study period for the TN treatment was 19.5 ± 2.11°C and for the HI treatment was 25.3 ± 4.78°C. For the HI treatment, the average daily room temperature during the periods from 08:00 to 19:00 h was 29.9 ± 0.68°C and from 20:00 to 07:00 h was 20.6 ± 1.14°C. These temperatures are close to the thermostat set point temperatures for those periods of the day, which were 30°C and 20°C, respectively (Figure 1a). The thermostat in the rooms used for the TN treatment was set at 24°C at the start of the study and reduced to 20 °C on d 15 of the study period and to 18°C on d 46. These temperature changes corresponded to the day after the pigs reached an average BW of approximately 35 and 70 kg, respectively. Average daily RT for the TN treatment from the start to d 14 was 23.4 ± 1.90 °C, from d 15 to 45 was 20.0 ± 0.70 °C, and from d 46 to the end of the study was 18.4 ± 1.30 °C. These temperatures were close to the target set point temperatures for this treatment during these periods. These results indicate that average RT levels were similar to the target levels for both RT treatments.

**Table 1.** Room temperature and relative humidity by RT treatment throughout the study period

Item	Room temperature^1^					
	Thermoneutral		High			
			08:00 to 19:00 h		20:00 to 07:00 h	
	Mean	SD	Mean	SD	Mean	SD
Temperature, °C						
Day of study						
0 to 14	23.4	1.90	29.5	0.59	20.1	0.84
15 to 45	20.0	0.70	29.9	0.55	20.4	0.75
46 to 119	18.4	1.30	30.0	0.73	20.8	1.27
Overall (d 0 to 119)	19.5	2.11	29.9	0.68	20.6	1.14
Relative humidity, %						
Day of study						
0 to 14	44.2	8.82	30.2	5.19	35.9	8.67
15 to 45	48.1	4.09	34.4	4.51	41.4	4.30
46 to 119	52.8	8.84	40.1	10.79	45.9	9.21
Overall (d 0 to 119)	50.4	8.45	37.3	9.66	43.4	8.85

Thermoneutral = thermostat set at constant temperature according to pig BW (24 °C from the start to 35 kg live BW, 20 °C from 35 to 70 kg live BW, and at 18 °C for the remainder of the study period); High = throughout the study period the thermostat was set at 30 °C from 08:00 to 19:00 h and at 20 °C from 20:00 to 07:00, with 1-h transition periods.

The relative humidity, averaged for the two rooms that were on each RT treatment, for the overall study period was greater for the TN than the HI treatment (50.4% vs. 40.4 %, respectively; Figure 1b). In addition, relative humidity increased over the 3 study periods (i.e., start to d 14, d 15 to 45, and d 46 to end) for both the TN (44.2 vs. 48.1 vs. 52.8 %, respectively, and the HI (33.1% vs. 37.9% vs. 43.0%, respectively) treatments. Relative humidity for the HI treatment was greater during the nighttime (20:00 to 07:00 h) than during the daytime (08:00 to 19:00 h; Table 1.1). The higher relative humidity levels for the TN than the HI treatment throughout the study period and for the HI treatment during the nighttime compared to the daytime corresponded to times of lower ambient temperatures (Figure 1a). Relative humidity is calculated as the water vapor present in the air expressed as a percentage of the total amount of water vapor required for saturation of the air. Warm air can hold more water vapor than cold air and, therefore, when the amount of water vapor in the air is constant, the relative humidity will be higher at lower air temperatures (Encyclopedia Britannica, 2013). Therefore, the differences in relative humidity both between the RT treatments and, also, at different times of the day for the HI treatment were most likely due to the temperature differences between and within treatments rather than a difference in the amount of water vapor in the air.

Relative humidity can influence growth performance, particularly at higher environmental temperatures. The response of pigs to environmental temperatures above the comfort zone is to increase evaporative heat loss by increasing respiration rate (Collier and Gebremedhin, 2015). Higher ambient relative humidity levels can reduce the effectiveness of this response. In support of this concept, Curtis (1983) and Huynh et al. (2005) have suggested that at an air temperature of 30 °C an increase in relative humidity of 18% is equivalent to an increase in air temperature of approximately 1 °C. However, there has been limited research to quantify the effect of relative humidity within the range observed in the current study (i.e., between approximately 30 and 50%) on the performance of growing-finishing pigs. Granier et al. (1998) compared the effects of relative humidity levels of 45, 60, and 75% in pigs of 25–105 kg BW kept at an ambient temperature of 28 °C and reported reductions in growth rate and feed intake with increasing relative humidity, with no effect on feed efficiency. In contrast, Morrison et al. (1966) found no effect of relative humidity levels of 45, 70, and 95% on weight gain or feed consumption of pigs reared from 30 to 100 kg BW at temperatures that were considered optimum for production (i.e., between 25 and 19 °C across the weight range evaluated). In addition, Renaudeau et al. (2011), in a meta-analysis of over 80 studies that investigated the effect of high ambient temperature in growing-finishing pigs, found no evidence of either an effect of relative humidity or an interaction between relative humidity and ambient temperature for growth performance. These authors indicated that this result was because the relative humidity in the majority of studies was below 80% and suggested that the effects of heat stress on feed intake are increased above 80% relative humidity. On the basis of this previous research, it is unlikely that the differences in relative humidity between the RT treatments in the current study influenced the observed growth responses, particularly as the levels were greater for the TN than the HI treatment.

There were no RT by DD interactions (*P* > 0.05) for any of the growth performance or water disappearance measurements taken during the study. Therefore, the main effects are presented and discussed separately.

**Figure 2.**
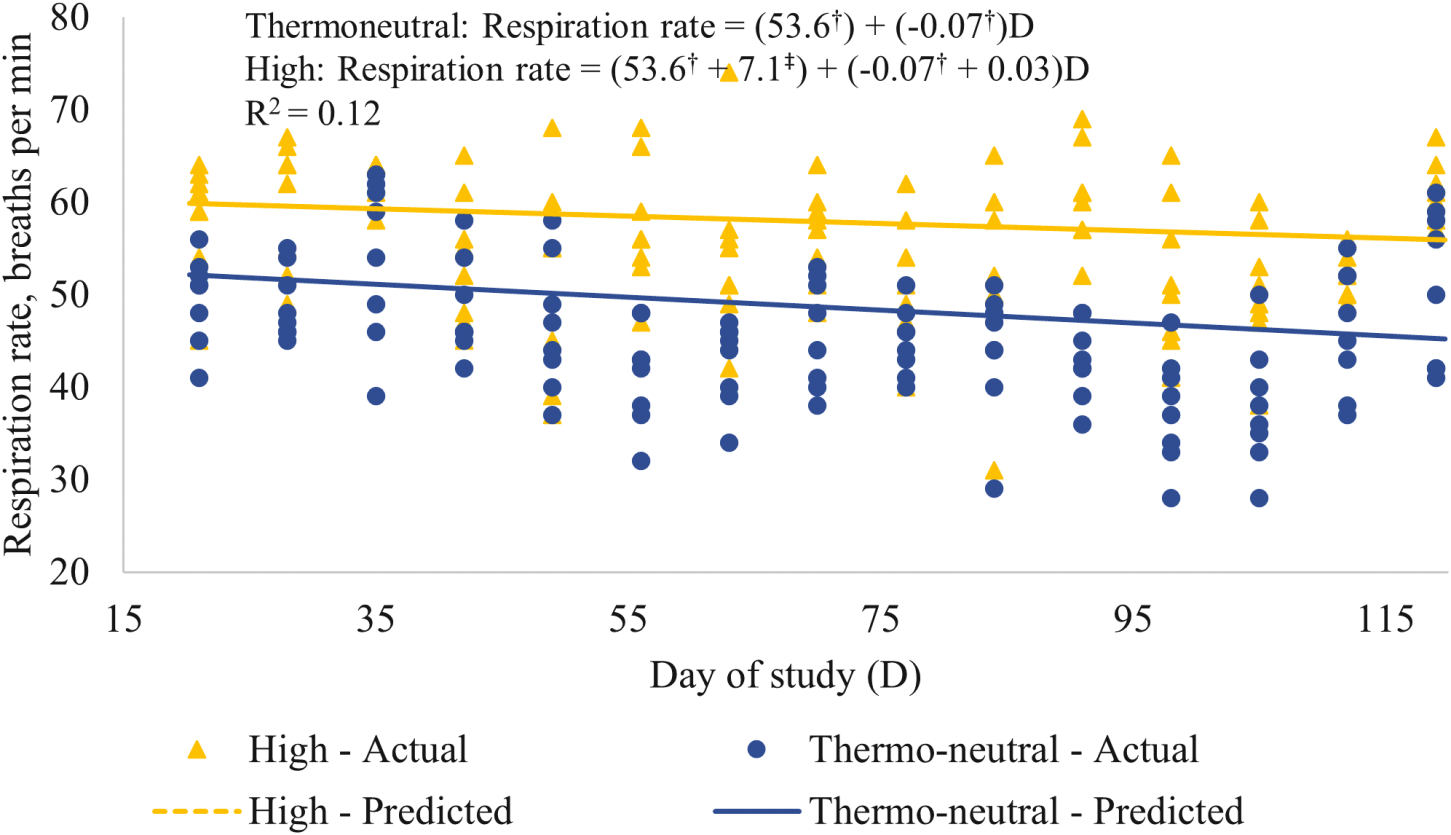
Regression relationships between respiration rate (breaths per minute) and day of study for the RT treatments. Thermoneutral = thermostat set at constant temperature according to pig BW (24 °C from the start to 35 kg live BW, 20 °C from 35 to 70 kg live BW, and at 18 °C for the remainder of the study period); High = throughout the study period the thermostat was set at 30 °C from 08:00 to 19:00 h and at 20 °C from 20:00 to 07:00, with 1-h transition periods. ^†^Indicates that the coefficient for the Thermoneutral treatment is different (*P* ≤ 0.05) to 0. ^‡^Indicates that the coefficient adjustment for the High treatment is different (*P* ≤ 0.05) to 0.

Least-squares means for the effect of RT on respiration rate for the days of the study that this measurement was taken and the average for the study period are presented in Table 2. Respiration rates were greater (*P* ≤ 0.05) for the HI than the TN treatment for the overall study period and for the majority of days. The regressions of respiration rate on day of study for the RT treatments are presented in Figure 2. The regressions for the two RT treatments differed in intercept (*P* ≤ 0.05), but not in slope (*P* > 0.05) indicating that respiration rate declined over the study period at a relatively similar rate for both RT. However, the predicted respiration rate was greater for the HI than the TN treatment at all times, with the treatment difference ranging from approximately 7 to 11 breaths/min (Figure 2). Increases in age and body size have been associated with decreasing respiration rates in pigs (Sipos et al., 2013) and in humans (Wallis et al., 2005).

**Table 2.** Least-squares means for the effect of RT treatment on respiration rate of growing-finishing pigs

Item	Room temperature^1^		SEM	*P*-value
	Thermoneutral	High		
Number of pigs^2^	16	16	–	–
Respiration rate (breaths/min)				
Measurement day of study				
21	49.6	58.4	1.83	0.001
28	49.0	59.9	3.86	<0.0001
35	54.1	62.4	3.31	0.01
42	48.4	53.6	4.50	0.16
49	46.6	52.3	5.91	0.09
56	41.4	57.4	2.36	<0.0001
63	42.6	54.4	3.04	0.01
70	45.9	56.4	3.20	0.003
77	44.9	51.0	2.49	0.09
84	44.0	52.0	3.14	0.08
91	42.9	60.5	2.39	<0.0001
98	37.6	51.9	2.73	0.001
105	37.9	50.5	2.29	0.001
112	46.3	51.5	3.07	0.24
119	51.1	61.4	3.93	0.0001
Overall (d 0 to 119)	45.5	55.6	1.69	<0.0001

Thermoneutral = thermostat set at constant temperature according to pig BW (24 °C from the start to 35 kg live BW, 20 °C from 35 to 70 kg live BW, and at 18 °C for the remainder of the study period); High = throughout the study periodthe thermostat was set at 30 °C from 08:00 to 19:00 h and at 20 °C from 20:00 to 07:00, with 1-h transition periods.

Measured on a randomly-selected sub-sample of 2 pigs from each of 4 pens per room on each day of measurement.

Increasing respiration rate is a response by the pig to increase evaporative heat loss from the respiratory tract at temperatures above the upper limit of the comfort zone. Huynh et al. (2005), in a study carried out in a respiration chamber with groups of 10 pigs between BW of 60 and 70 kg, found that respiration rate was the first physiologic indicator of a heat stress response. The results of that study indicated that respiration rate increased at temperatures above 23.4 °C (for pigs kept at 50% relative humidity). However, in the study of Huynh et al. (2005) the increase in respiration rate was approximately 15 breaths/min for every 1 °C increase above that temperature, which is substantially greater than the difference between the RT treatments in the current study, which was equivalent to an increase of between 1 and 2 breaths/min/°C. Other studies have also shown much greater increases in respiration rate with increasing temperature than in the present study. Pearce et al. (2013) found that, for pigs of 35 kg BW, respiration rate was double at 35 °C compared to that at 20 °C (106 and 54 breaths/min, respectively). Brown-Brandl et al. (1998) found that, for pigs of 83.5 kg BW, respiration rate increased exponentially from approximately 20 to in excess of 100 breaths/min with increases in environmental temperature from 18 to 32 °C. However, in all of those studies the pigs were first exposed to the higher temperatures at the start of the experimental period, without an acclimation phase. Studies in which the pigs have been exposed to higher temperatures for an extended period of time, including the current experiment, have generally shown more modest increases in respiration rate with increasing temperature (Lopez et al., 1991). In addition, a number of other factors can have a major impact on respiration rates including the method of measurement (Brown-Brandl et al., 2001) and the time of day at which the measurement is taken (Christon, 1988; Lopez et al., 1991). In the current study, the purpose of measuring respiration rate was to confirm that the temperature regime for the HI treatment was effective at eliciting a heat stress response; the consistently higher respiration rate for this treatment than the TN treatment across the study period validates that this was the case.

Least-squares means for the effect of RT treatment on BW at the start and end of test and at d 14 and 56 of the study and for growth performance for the overall study period and the interim time periods are presented in Table 3. There was no effect (*P* > 0.05) of RT treatment on average BW at the start and d 14 of the study period (Table 3). However, BW on d 56 and at the end of the study were lower (*P* ≤ 0.05) for the HI than the TN treatment (by 2.1 and 8.3 kg, respectively). During the first 2 wk of the study, ADG and ADFI were not different (*P* > 0.05) between RT; for other time periods and the overall study period pigs on the HI treatment had lower (*P* ≤ 0.05) ADG and ADFI than those on the TN treatment. For example, for the overall study period ADG was 0.07 kg (7.4 %) and ADFI was 0.2 kg (7.2 %) lower for the HI compared to the TN treatment. However, there was no effect (*P* > 0.05) of RT on G:F ratio in any period of the study (Table 3).

**Table 3.** Least-squares means for the effect of RT treatment on the growth performance and water disappearance of growing-finishing pigs

Item	Room Temperature^1^		SEM	*P*-value
	Thermoneutral	High		
Number of rooms	2	2	–	–
Number of pens	16	16	–	–
Number of pigs	158	158	–	–
Average body weight, kg				
Start (0 d)	26.1	26.0	0.70	0.86
14 d	35.7	35.6	0.63	0.73
56 d	77.9	75.8	2.09	0.01
End (119 d)	135.0	126.7	0.73	< 0.0001
Average daily gain, kg				
0 to 14 d	0.70	0.69	0.025	0.78
14 to 56 d	0.98	0.93	0.028	0.001
0 to 56 d	0.92	0.88	0.008	0.002
56 to 119 d	1.03	0.87	0.029	0.003
Overall (d 0 to 119)	0.95	0.88	0.024	< 0.0001
Average daily feed intake, kg				
0 to 14 d	1.49	1.48	0.045	0.56
14 to 56 d	2.29	2.18	0.069	0.01
0 to 56 d	2.09	2.00	0.052	0.01
56 to 119 d	3.51	3.18	0.074	< 0.0001
Overall (day 0 to 119)	2.76	2.56	0.087	< 0.0001
Gain:Feed, kg:kg				
0 to 14 d	0.458	0.460	0.0156	0.81
14 to 56 d	0.428	0.426	0.0066	0.64
0 to 56 d	0.437	0.436	0.0048	0.75
56 to 119 d	0.281	0.279	0.0057	0.52
Overall (day 0 to 119)	0.357	0.355	0.0052	0.48
Average daily water disappearance, L				
0 to 14 d	5.13	5.34	0.476	0.24
14 to 56 d	5.63	6.64	0.411	< 0.0001
0 to 56 d	5.51	6.32	0.443	0.0001
56 to 119 d	6.44	7.65	0.370	< 0.0001
Overall (d 0 to 119)	5.96	6.96	0.363	< 0.0001
Water:Feed, L:kg				
Start to 14 d	3.19	3.33	0.191	0.14
14 to 56 d	2.30	2.81	0.255	< 0.0001
0 to 56 d	2.47	2.92	0.228	< 0.0001
56 to 119 d	1.70	2.39	0.107	< 0.0001
Overall (d 0 to 119)	2.12	2.70	0.139	< 0.0001

Thermoneutral = thermostat set at constant temperature according to pig BW (24 °C from the start to 35 kg live BW, 20 °C from 35 to 70 kg live BW, and at 18 °C for the remainder of the study period); High = throughout the study periodthe thermostat was set at 30 °C from 08:00 to 19:00 h and at 20 °C from 20:00 to 07:00, with 1-h transition periods.

**Figure 3.**
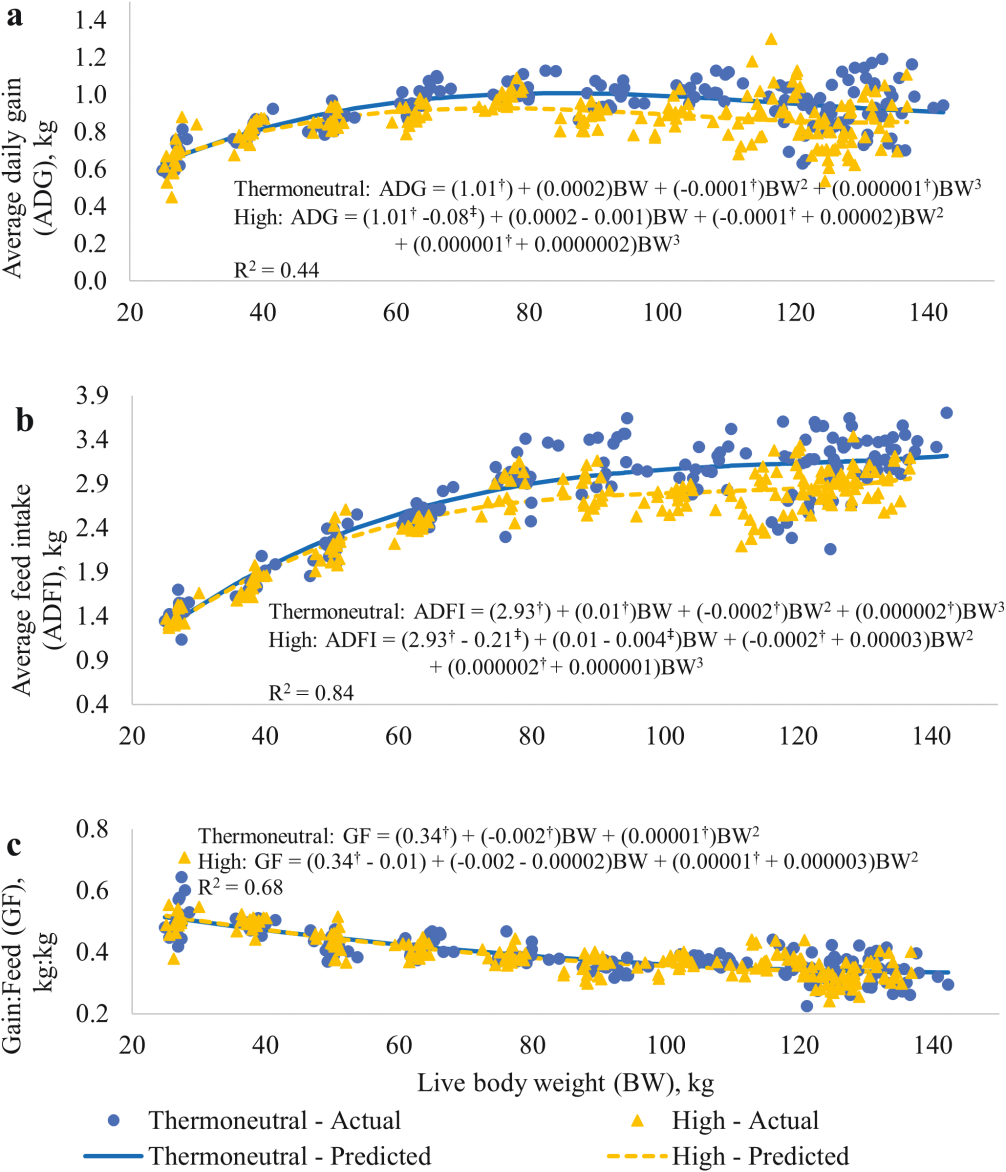
Regression relationships between growth performance and average pig live weight for the RT treatments. (**a**) Average daily gain. (**b**) Average daily feed intake. (**c**) Gain:feed ratio. All regressions were centered at 82.8 kg live BW. Thermoneutral = thermostat set at constant temperature according to pig BW (24 °C from the start to 35 kg live BW, 20 °C from 35 to 70 kg live BW, and at 18 °C for the remainder of the study period); High = throughout the study period the thermostat was set at 30 °C from 08:00 to 19:00 h and at 20 °C from 20:00 to 07:00, with 1-h transition periods. ^†^Indicates that the coefficient for the thermoneutral treatment is different (*P* ≤ 0.05) to 0. ^‡^Indicates that the coefficient adjustment for the High treatment is different (*P* ≤ 0.05) to 0.

The regression relationships between ADG, ADFI, and G:F and average pen BW for the RT treatments are presented in Figure 3a–c, respectively. The relationship between both ADG and ADFI and BW was cubic; the relationship between G:F and BW was quadratic. For ADG, the intercept, quadratic, and cubic coefficients for BW for the TN treatment were different (*P* ≤ 0.05) to 0 (Figure 3a). The intercept for the HI treatment was lower (*P* ≤ 0.05) than that of the TN treatment. For both RT treatments, ADG increased with BW up to an average pen BW of approximately 70 kg and then declined (Figure 1a). The difference in ADG between the RT treatments was generally greater at higher average pen BW. For the regression relationship between ADFI and average pen BW, the intercept, and the three coefficients for BW in the equation for the TN treatment were different (*P* ≤ 0.05) from 0, and the intercept and linear coefficient were different (*P* ≤ 0.05) for the HI compared to the TN treatment (Figure 3b). Average daily feed intake increased across the range of average pen BW, however, the rate of increase declined with BW (Figure 3b). In addition, the difference in ADFI between RT generally increased as BW increased. For G:F, the intercept, and linear and quadratic coefficients for BW in the regression equation for the TN treatment were different (*P* ≤ 0.05) to 0; however, there was no difference (*P* > 0.05) between the coefficients for the HI compared to the TN treatment (Figure 3c). Gain:feed ratio decreased across the range of BW at a similar rate in both RT treatments.

The decrease in ADG and ADFI for the overall study period for the HI compared to the TN treatment was 7.4 and 7.2%, respectively (Table 3). The similarity of these decreases and the absence of any treatment effect on G:F ratio suggests that the reduced growth rate for pigs exposed to the higher temperatures was largely due to the reduction in feed intake rather than any effect on the efficiency with which feed was converted into live weight gain. This result is similar to that found in most previous research. For example, Le Bellego et al. (2002) showed that keeping individually-housed barrows at constant ambient temperatures of 29 compared to 22 °C between 27 and 100 kg BW reduced ADG and ADFI but had no effect on feed conversion efficiency. Renaudeau et al. (2011), based on a meta-analysis of studies investigating the effect of high ambient temperature on growth performance of growing-finishing pigs that were published between 1960 and 2009, suggested that the feed conversion ratio of a 50 kg pig (the average weight of pigs used across studies) did not change with increases in ambient temperature between 20 and 30 °C. Above this temperature, there was a small deterioration in feed conversion ratio, suggesting that more severe heat stress may have a negative effect on feed efficiency.

Comparing the magnitude of the reduction in growth performance due to high temperature in the current experiment with that of published studies is complicated because most previous studies have used constant rather than cyclic temperature treatments. In the meta-analysis of Renaudeau et al. (2011), only 16% of the papers addressing the effects of high ambient temperature on growth performance used cyclic temperature regimes. Also, there is disagreement in the literature on the relative effects of constant compared to cyclic temperatures when the average temperatures of the two regimes are the same. Xin and DeShazer (1991) found that pigs exposed to a cyclic temperature regime with a range of 7 °C around an average of 30.8 °C had similar ADFI and ADG to those kept at a constant temperature with the same average. However, in that study, a range of 16.6 °C around the same average resulted in a substantial reduction in growth performance. Quiniou et al. (2000) compared constant temperatures of either 24 or 28 °C with cyclic temperatures with ranges of 3, 6, and 9 °C around those constant temperatures. Compared to the constant temperatures, a 3 °C range had no effect on ADFI and ADG, but both of these were reduced when the range in temperature was either 6 or 9 °C. However, the treatment differences were relatively small. For example, at the mean temperature of 28 °C, a range of 9 °C reduced ADFI and ADG by 1.0 and 1.9%, respectively, compared to the constant temperature. In addition, Morrison (1975) found that ADG, ADFI, and feed conversion ratio were similar for growing-finishing pigs exposed to a cyclic temperature regime with a range of 10 °C compared to a constant temperature treatment with the same average.

In the current study, the measured average temperatures for the HI treatment for the nighttime and daytime were 20.6 and 29.9 °C, respectively (Table 1). Based on the literature discussed above, it would seem probable that the performance of the pigs on the HI treatment would be similar to that of pigs exposed to a constant temperature of 25.3 °C (i.e., the average of the nighttime and daytime temperatures). This equates to a difference between the average measured temperatures for the TN, which was 19.5 °C (Table 1), and HI treatments of 5.8 °C. The reduction in ADG and ADFI for the overall study period for the HI compared to the TN treatment was 0.07 and 0.2 kg, respectively (Table 3) which would equate to reductions of approximately 12 and 34 g/°C, respectively (Table 3). Several studies that have been carried out with growing-finishing pigs that have evaluated the effect of increasing temperatures across the range from 20 to 30 °C have reported relatively greater changes in ADG and ADFI than in the current study. For example, Le Bellego et al. (2002) reported reductions in ADG and ADFI for barrows (27–100 kg BW) between temperatures of 22 and 29 °C of approximately 20 and 55 g/°C, respectively. Similarly, Nienaber et al. (1987) reported that as temperature increased from 20 to 30 °C, ADG, and ADFI declined by 30 and 70 g/°C, respectively. However, as discussed previously, these studies evaluated the effects of constant temperatures which were also higher than the average temperature of the HI treatment in the current study. In addition, a number of studies have shown that the relationship between ambient temperature and growth is curvilinear, with the negative effects on ADG and ADFI increasing with temperature. The meta-analysis of published studies carried out by Renaudeau et al. (2011) estimated that for a pig weighing 50 kg BW the instantaneous rate of decline in ADG at 25 and 30 °C was approximately 11 and 25 g/°C, respectively, and the instantaneous rate of decline in ADFI was approximately 32 and 56 g/°C, respectively, at the same temperatures. The instantaneous rates of decline for ADG and ADFI at 25 °C reported by Renaudeau et al. (2011) were relatively similar to the rates for the HI treatment (average temperature of 25.3 °C) in the current study discussed above. This suggests that the decrease in ADG and ADFI observed in the present study was in line with what would be expected based on previous research given the differences in temperature for the temperature treatments compared.

Least-squares means for the effect of RT on ADWD and water:feed ratio are presented in Table 3. There was no effect (*P* > 0.05) of RT on either measurement during the first 2 wk of the study period. However, for the subsequent and the overall study periods ADWD and water:feed ratio were greater (*P* ≤ 0.05) for the HI than the TN treatment. The difference between the treatments for these two variables for the overall study period was 1.0 L/d and 0.58 L:kg, respectively, or 16.8 and 27.4%, respectively, of the mean of the TN treatment (Table 3).

**Figure 4.**
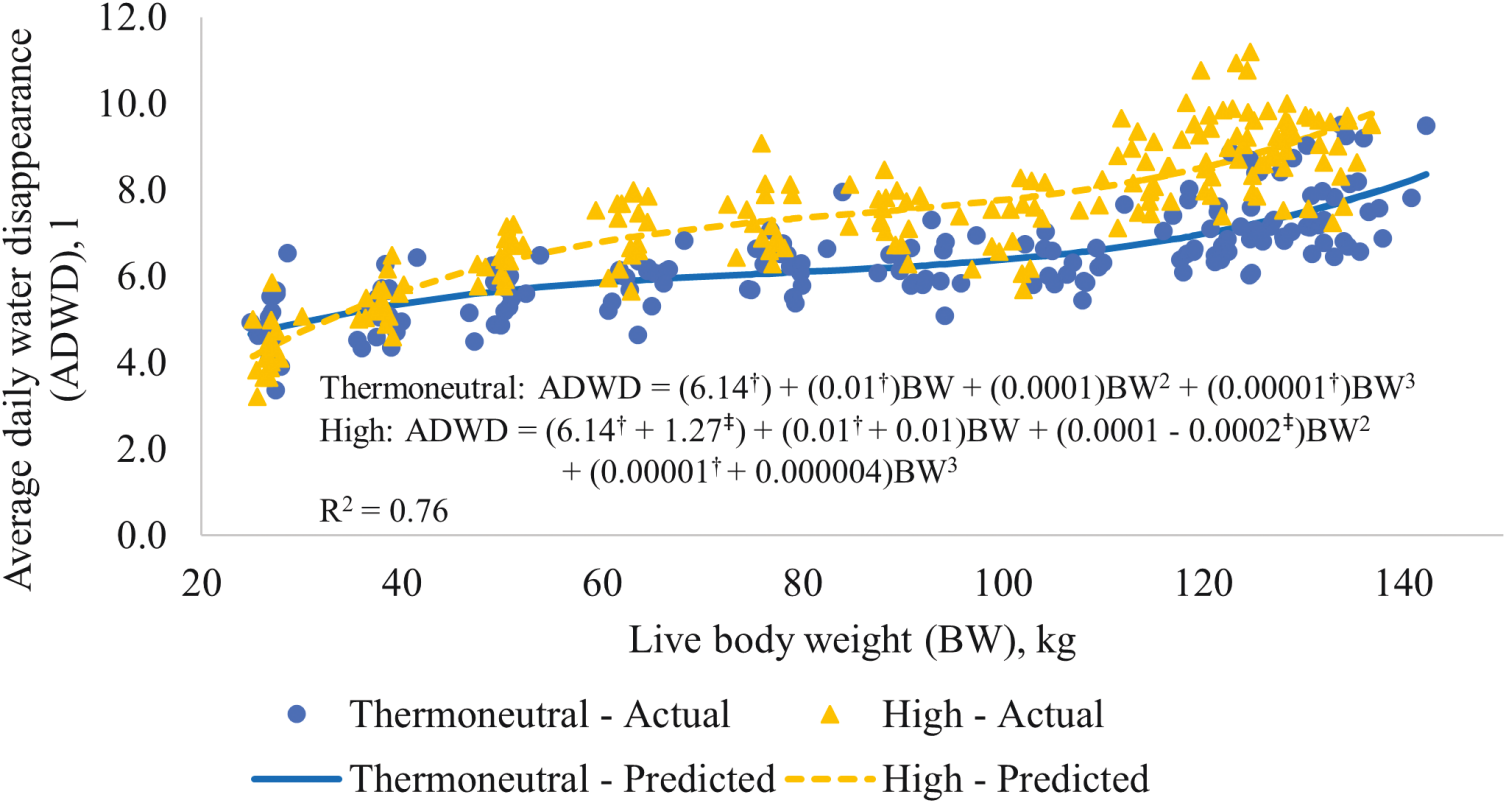
Regression relationships between average daily water disappearance and average pig live body weight for the RT treatments. Regressions centered at 82.8 kg live BW. Thermoneutral = thermostat set at constant temperature according to pig BW (24 °C from the start to 35 kg live BW, 20 °C from 35 to 70 kg live BW, and at 18 °C for the remainder of the study period); High = throughout the study period the thermostat was set at 30 °C from 08:00 to 19:00 h and at 20 °C from 20:00 to 07:00, with 1-h transition periods. ^†^Indicates that the coefficient for the thermoneutral treatment is different (*P* ≤ 0.05) to 0. ^‡^Indicates that the coefficient adjustment for the High treatment is different (*P* ≤ 0.05) to 0.

The regression relationships between ADWD and average pen BW for the two RT treatments are presented in Figure 4. For the TN treatment, the intercept, and the linear, and cubic coefficients for BW were different (*P *≤ 0.05) from 0. The intercept and quadratic coefficient for the HI treatment were different (*P* ≤ 0.05) to those for the TN treatment. The difference in ADWD between the treatments was relatively limited in the early part of the study, but generally increased with increasing BW (Figure 4).

Xin and DeShazer (1991) found no effect of constant and cyclic temperature regimes on daily water disappearance when these regimes had the same mean temperature. This suggests that the effect of the cyclic temperature regime on water disappearance in the current study would be similar to that of a constant temperature that was the same as the mean temperature for the cyclic regime. As previously discussed, the difference between the average daily ambient temperature for the TN and HI treatments for the overall study period was 5.8 °C. In addition, the increase in ADWD and water:feed ratio for the overall study period for the HI compared to the TN treatment were 1.0 L and 0.58 L:kg, respectively (Table 3). Therefore, this increase in ADWD for the HI treatment was equivalent to 0.17 L/d or 2.9% of the mean of the TN treatment for every 1 °C increase in temperature. Similarly, the increase in water:feed ratio for the HI relative to the TN treatment was equivalent to 0.10 L/kg or 4.7% every 1 °C increase in temperature.

A limited number of studies have reported on the effect of high ambient temperatures on water consumption and water:feed ratio and these have produced variable results (Mount et al., 1971). This is not surprising given the variation between studies in a number of factors that can have a major impact on either water and/or feed intake levels, including the weight range over which the study was conducted, the temperatures compared, and the DD used. Most studies have shown that both water disappearance and water:feed ratio generally increase with increasing ambient temperature. However, reported values for water disappearance and water:feed ratio and for the rates of increase with ambient temperature have varied widely between studies. Lopez et al. (1991), in a 21-d study carried out with pigs between 90 and 110 kg BW kept at either a constant ambient temperature of 20 °C or at a cyclic temperature with a mean of 28.5 °C, reported daily water disappearance for the 2 treatments of approximately 7 and 10 L/pig, respectively, equivalent to an increase of approximately 0.35 L/d/°C or 5%/°C. In that study, water:feed ratio for the cyclic 28.5 °C treatment was greater than for the constant 20 °C treatment, with means of 3.6 and 2.1 L:kg, respectively, equivalent to an increase of 0.18 L:kg/°C or 8.4 %/°C. Nienaber et al. (1987), in a study carried out with pigs between 45 and 90 kg BW, reported that the water disappearance of pigs kept at 25 °C was 2.4 L/d greater than for those kept at 20 °C, which is equivalent to an increase in water disappearance of 0.48 L/d/°C. The values for changes in water disappearance with increasing ambient temperature in the studies of Lopez et al. (1991) and Nienaber et al. (1987) are greater than those found in the current study. However, Massabie et al. (1996) found that water:feed ratio for pigs of 25–105 kg BW increased by approximately 0.13 L:kg/°C between temperatures of 20 and 28 °C which is intermediate between the results of the current and the other studies.

The studies discussed above have shown that water disappearance and water:feed ratio increase at temperatures above 20 °C. In contrast, Huynh et al. (2005) evaluated the impact of keeping pigs at temperatures between 16 and 32 °C at 50% relative humidity and found that water:feed ratio was constant up to 25.4 °C (at approximately 2.4 L:kg) and subsequently increased by 0.22 L:kg/°C. However, in that study, the pigs were exposed to each temperature for only 1 d, which could have contributed to the different responses observed compared to other studies. This discussion highlights the lack of consensus in the literature regarding the relationship between increasing ambient temperature and water consumption.

Least-squares means for the effect of DD treatment on growth performance, water disappearance, and water:feed ratio are presented in Table 4. There was no effect (*P* > 0.05) of DD on average BW, ADG, or ADFI. Gain:Feed ratio was greater (*P* ≤ 0.05) for the Nipple than the Cup treatment during the first 14 d of the study period. However, the difference between the DD treatments was small and there was no difference (*P* > 0.05) between the DD for G:F ratio for any of the subsequent time periods or overall (Table 4). Average daily water disappearance was greater (*P* ≤ 0.05) for the Nipple compared to the Cup treatment in every period of the study and overall (Table 4). In addition, water:feed ratio was greater (*P* ≤ 0.05) for Nipple than Cup drinkers up to d 56 of the study, and for the overall study period, but not (*P *> 0.05) for the period from d 56 to the end of the study (Table 4).

**Table 4.** Least-squares means for the effect of Drinker Design treatment on the growth performance and water disappearance of growing-finishing pigs

Item	Drinker Design^1^		SEM	*P*-value
	Nipple	Cup		
Number of pens	16	16	–	–
Number of pigs	157	159	–	–
Average body weight, kg				
Start (0 d)	26.5	26.3	0.51	0.49
14 d	35.8	35.5	0.63	0.33
56 d	77.3	77.1	1.70	0.77
End (119 d)	131.2	130.6	1.29	0.74
Average daily gain, kg				
Start to 14 d	0.70	0.69	0.025	0.51
14 to 56 d	0.95	0.96	0.028	0.73
Start to 56 d	0.90	0.90	0.016	0.95
56 to 119 d	0.95	0.95	0.026	0.80
Overall (d 0 to 119)	0.92	0.92	0.016	0.87
Average daily feed intake, kg				
Start to 14 d	1.47	1.50	0.045	0.18
14 to 56 d	2.23	2.25	0.069	0.72
Start to 56 d	2.04	2.06	0.053	0.53
56 to 119 d	3.36	3.32	0.093	0.44
Overall (d 0 to 119)	2.68	2.67	0.062	0.77
Gain:Feed, kg:kg				
Start (0 d) to 14 d	0.466	0.452	0.0156	0.02
14 to 56 d	0.427	0.427	0.0066	0.88
Start to 56 d	0.438	0.435	0.0047	0.19
56 to 119 d	0.278	0.282	0.0056	0.25
Overall (d 0 to 119)	0.356	0.356	0.0053	0.96
Average daily water disappearance, L				
Start to 14 d	5.54	4.93	0.476	0.001
14 to 56 d	6.38	5.90	0.411	0.01
Start to 56 d	6.15	5.63	0.280	0.01
56 to 119 d	7.17	6.79	0.256	0.02
Overall (d 0 to 119)	6.64	6.19	0.258	0.01
Water:Feed, L:kg				
Start to 14 d	3.45	3.06	0.191	0.0003
14 to 56 d	2.65	2.46	0.255	0.03
Start to 56 d	2.85	2.62	0.140	0.01
56 to 119 d	2.09	2.01	0.115	0.23
Overall (d 0 to 119)	2.47	2.32	0.122	0.04

Nipple = the water source within the pen was a nipple-type drinker; Cup = the water source within the pen was a cup-type drinker.

For the overall study period, ADWD was 7.3% greater for Nipple compared to Cup drinkers. However, there was no effect of DD on overall growth performance which suggests that the higher water disappearance for the nipple drinkers was mainly due to increased water wastage. Most other studies that have evaluated water disappearance from nipples compared to cups or bowls have also shown that levels are greater for nipple drinkers. Alvarez-Rodriguez et al. (2013) found that water disappearance for pigs between 20 and 100 kg BW was greater for two designs of nipple-type drinkers than for two designs of bowls. However, in that study, water disappearance levels for the nipple-type drinkers (8.2 and 9.7 L/d) and the difference between the nipples and the bowls (19–73%) were considerably greater than in the current study. Similarly, Brumm and Heemstra (1999) , in a study carried out with pigs between 17 and 115 kg BW, reported that water disappearance was 33% greater for a swing-type nipple drinker than for a bowl drinker. However, the absolute levels of water disappearance were lower in that study (3.8 and 5.0 L/d for the bowl and nipple drinkers, respectively) than in the current experiment. In contrast, Tavares et al. (2014) carried out a study on 15 commercial growing-finishing facilities with pigs from approximately 25–127 kg BW and reported that ADWD for nipple-type drinkers was approximately 19% lower than for bite-ball nipple-type drinkers, and approximately 16% lower compared to bowls. However, in that study, each building was fitted with only one of the DDs and water disappearance was only measured on the single water line that supplied each building. Therefore, it is possible that a number of factors that differed between the buildings used could have confounded those results.

Differences between studies in both the absolute level of water disappearance and in the magnitude of the difference between nipples and bowls or cups are likely to be due, in part, to differences in the level of water wastage. Li et al. (2005) found that the average water wastage from nipple drinkers was approximately 26% of water disappearance. However, there was considerable variation in the level of wastage (from 15% to 42% of water disappearance) depending on factors such as water flow rate and height of the nipple drinkers. Li et al. (2005) also reported on a study with growing-finishing pigs (32 to 105 kg BW) that showed that water disappearance from bowls was approximately 10% less than for nipples, which is relatively similar to the findings of the current study. However, the nipple drinkers in that study were not adjusted for the increases in the height of the pigs over the study period. When Li et al. (2005) adjusted the height of the nipple drinkers for the increasing size of pigs using the same protocol as in the current study, water disappearance was not different between bowls and nipples. In the current study, even with adjustments to the heights of the nipples as the pigs increased in size, water disappearance was still greater than for the cup drinkers. This highlights the considerable variability between published studies in the relative effect of DDs on water disappearance and wastage. This variability is to be expected given the many factors that differed between studies that can affect water wastage and disappearance, including the specific design of drinkers compared, the weight ranges over which the studies were carried out, the water flow rates to the drinkers (Li et al., 2005), and the group sizes used (Turner at al., 1999).

In the current study, the water:feed ratio was 6.5% greater for the Nipple than the Cup DD, which is similar to the relative difference between these treatments for water disappearance. As previously discussed, the greater water:feed ratio for the nipple drinkers was most likely the result of differences in water wastage between the DD. This suggests that the relationship between feed intake and the actual water consumption by the pigs was relatively similar for the two DD treatments. The NRC (2012) suggested that the minimum water requirement for pigs between 20 and 90 kg BW was approximately 2 kg water/kg feed and that the voluntary water intake of growing pigs given ad libitum access to feed was approximately 2.5 kg of water for each kg of feed. In the current study, the water:feed ratios for both DD treatments were within this range (Table 4) and are relatively similar to values reported in other studies. For example, Li et al. (2005) reported that the water:feed ratio of growing and finishing pigs given access to nipples was 2.43 and 2.13 L:kg, respectively. Brumm and Heemstra (1999) found water:feed ratios of 1.89 and 2.41 L:kg for growing-finishing pigs given access to either bowl or swing-type nipple drinkers, respectively, with this difference between DDs being largely the result of differences in water disappearance rather than any effect on feed intake. This suggests that variation in water:feed ratio between studies and DD treatments within studies is mainly due to differences in water wastage. Consequently, the same factors previously identified that influence water wastage, such as water flow rates to the drinkers, will also influence water:feed ratios. However, other factors can also have an impact. For example, Shaw et al. (2006), in a study carried out in metabolism cages with pigs with an initial BW of 34 kg, found that water:feed ratio was greater for diets with excess crude protein levels compared to diets with lower, more typical levels of crude protein. The authors reported that water wastage in that study was very low (<1%), which suggests that the differences between the diets in water:feed ratio reflected a true difference in the relationship between feed and water intake levels.

**Figure 5.**
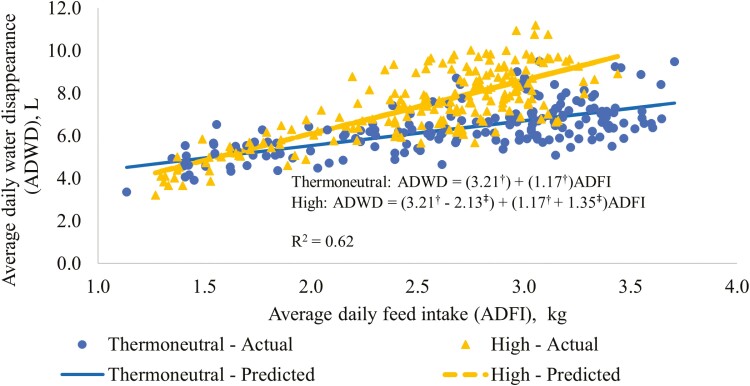
Regression relationships between average daily water disappearance and average daily feed intake for the RT treatments. Thermoneutral = thermostat set at constant temperature according to pig BW (24 °C from the start to 35 kg live BW, 20 °C from 35 to 70 kg live BW, and at 18 °C for the remainder of the study period); High = throughout the study period the thermostat was set at 30 °C from 08:00 to 19:00 h and at 20 °C from 20:00 to 07:00, with 1-h transition periods. ^†^Indicates that the coefficient for the thermoneutral treatment is different (*P* ≤ 0.05) to 0. ^‡^Indicates that the coefficient adjustment for the High treatment is different (*P* ≤ 0.05) to 0.

Since the RT treatment had relatively large effects on both feed and water disappearance, the relationship between these two measurements was further investigated by evaluating the regression relationships between ADWD and ADFI for the two RT treatments, and these results are presented in Figure 5. Linear regressions gave the best fit to these data. The intercept and slope for the TN treatment were different (*P* ≤ 0.05) from 0. In addition, the intercept was lower (*P* ≤ 0.05), and slope was greater (*P* ≤ 0.05) for the HI compared to the TN treatment (Figure 5). At low ADFI, the predicted ADWD was lower for the HI than the TN treatment, but the treatment difference was limited. However, the rate of increase in ADWD with increasing ADFI for the HI treatment was more than double that for the TN treatment (2.52 vs. 1.17 L/kg, respectively; Figure 5). This suggests that heat stress can cause a change in the relationship between feed intake and water disappearance. Further research would be needed to determine the dynamics of changes in feed and water intake at high environmental temperatures. As with other aspects of water consumption, the relationship with feed intake is poorly understood and merits further study.

This study evaluated the effects of ambient temperatures typical of summer conditions in the Midwest of the U. S. Results suggested that the reduction in growth rate associated with elevated temperatures was due to reduced feed intake rather than because of any effect on the efficiency of use of feed for live weight gain. In addition, the higher level of water disappearance for the nipple compared to the cup-type drinkers evaluated in the study was most likely the result of increased water wastage rather than any difference in the amount of water consumed by the pigs.

## References

[CIT0001] Alvarez-Rodriguez, J., B. Hermida, J. Parera, H. Morazin, J. Balcells, and D. Babot. 2013. The influence of drinker device on water use and fertilizer value of slurry from growing-finishing pigs. Anim. Prod. Sci. 53:328–334. doi: 10.1071/AN12136.

[CIT0002] Brown-Brandl, T. M., R. A. Eigenberg, J. A. Nienaber, and S. D. Kachman. 2001. Thermoregulatory profile of a newer genetic line of pigs. Livest. Prod. Sci. 71:253–260. doi: 10.1016/S0301-6226(01)00184-1.

[CIT0003] Brown-Brandl, T. M., J. A. Nienaber, and L. W. Turner. 1998. Acute heat stress effect on heat production and respiration rate in swine. Trans. ASAE 41(3):789–793. doi: 10.13031/2013.17216.

[CIT0004] Brumm, M. C., and J. Heemstra. 1999. Impact of drinker type on pig performance, water use and manure production. 1999 Nebraska Swine Reports EC99-219:49–50.

[CIT0005] Campos, P. H. R. F., N. Le Floc’h, J. Noblet, and D. Renaudeau. 2017. Physiological responses of growing pigs to high ambient temperature and/or inflammatory challenges. Revista Bras. Zoo. 46:537–544. doi: 10.1590/S1806-92902017000600009.

[CIT0006] Christon, R. 1988. The effect of tropical ambient temperature on growth and metabolism in pigs. J. Anim. Sci. 66:3112–23. doi: 10.2527/jas1988.66123112x 3230073

[CIT0007] Collier, R. J., and K. G. Gebremedhin. 2015. Thermal biology of domestic animals. Ann. Rev. Anim. Biosci. 3:513–532. doi: 10.1146/annurev-animal-022114-110659. 25387108

[CIT0008] Curtis, S. E. 1983. Environmental Management in Animal Agriculture. Iowa State Univ. Press, Ames.

[CIT0009] Encyclopedia Britannica. 2013. “Humidity”. Britannica, the Editors of Encyclopaedia. Retrieved on 22 November 2021, from https://www.britannica.com/science/humidity. Accessed November 22, 2021.

[CIT0010] Granier, R., P. Massabie, and A. Bouby. 1998. Incidence du taux d’humidité relative de l’air ambiant sur les performances zootechniques du porc à l’ emgrais élevé à 28oC. Journées de la Recherche Porcine en France 30:331–336.

[CIT0011] 2010. Guide for Care and Use of Agricultural Animals in Research and Teaching. 3rd ed. American Dairy Science Association, American Society of Animal Science, and Poultry Science Association.

[CIT0012] Huynh, T. T. T., A. J. A. Aarnink, M. W. A. Verstegen, W. J. J. Gerrits, M. J. W. Heetkamp, B. Kemp, and T. T. Canh. 2005. Effects of increasing temperatures on physiological changes at different humidities. J. Anim. Sci. 83:1385–96. doi: 10.2527/2005.8361385x 15890816

[CIT0013] Illinois Climate Network. 2021. Water and Atmospheric Resources Monitoring Program. Illinois State Water Survey, 2204 Griffith Drive, Champaign, IL 61820–7495. doi: 10.13012/J8MW2F2Q.

[CIT0014] Le Bellego, L., J. van Milgen, and J. Noblet. 2002. Effect of high temperature and low-protein diets on the performance of growing-finishing pigs. J. Anim. Sci. 80:691–701. doi: 10.2527/2002.803691x. 11890404

[CIT0015] Li, Y. Z., L. Chenard, S. P. Lemay, and H. W. Gonyou. 2005. Water intake and wastage at nipple drinkers by growing-finishing pigs. J. Anim. Sci. 83:1413–22. doi: 10.2527/2005.8361413x 15890820

[CIT0016] Littell, R. C., G. A. Milliken, W. W. Stroup, and R. D. Wolfinger. 1996. SAS systems for mixed models. SAS Institute, Cary, NC.

[CIT0017] Little, S. B., H. K. Crabb, A. P. Woodward, G. F. Browning, and H. Billman-Jacobe. 2019. Review: water medication of growing pigs: sources of between animal variability in systemic exposure to antimicrobials. Animal. 13:3031–3040. doi: 10.1017/S1751731119001903. 31475656

[CIT0018] Lopez, J., G. W. Jesse, B. A. Becker, and M. R. Ellersieck. 1991. Effects of temperature on the performance of finishing swine: I. Effects of a hot, diurnal temperature on average daily gain, feed intake, and feed efficiency. J. Anim. Sci. 69:1843–9. doi: 10.2527/1991.6951843x 2066294

[CIT0019] Massabie, P., R. Granier, and J. L. Dividich. 1996. Influence de la temperature ambiante sur les performances zootechniques du porc a` l’engrais alimente´ ad libitum. J. Rech. Porcine Fr. 28:189–194.

[CIT0020] Morrison, S. R., H. Heitman, T. E. Bond, and P. Finn-Kelcey. 1966. The influence of humidity on growth and feed utilization of swine. Int. J. Biometeorol. 10:163–168. doi: 10.1007/BF01426862. 6009553

[CIT0021] Morrison, S. R., H. Heitman Jr., and R L. Givens. 1975. Effect of diurnal air temperature cycles on growth and food conversion in pigs. Anim. Prod. 20:287–291. doi: 10.1017/S0003356100035297.

[CIT0022] Mount, L. E., C. W. Holmes, W. H. Close, S. R. Morrison, and I. B. Start. 1971. A note on the consumption of water by the growing pig at several environmental temperatures and levels of feeding. Anim. Sci. 13:561–563. doi: 10.1017/S000335610001076X.

[CIT0023] Nienaber, J. A., G. L. Hahn, R. A. Eigenberg, R. L. Kothals, J. T. Yen, and D. L. Harris. 1997. Genetic and heat stress interaction effects on finishing swine. Proc. of the 5th Int. Symp., Bloomington, MN, USA. Livest. Envir. 5(2):1017–1023.

[CIT0024] Nienaber, J. A., G. L. Hahn, and J. T. Yen. 1987. Thermal environment effects on growing-finishing swine. I. Growth, feed intake and heat production. Trans. ASAE (Am. Soc. Agric. Eng.) 30:1772–1775. doi: 10.13031/2013.30635.

[CIT0025] NRC. 2012. Nutrient Requirements of Swine, 11th ed. National Academy Press, Washington, DC.

[CIT0026] Patience, J. F. 2012. Water in swine nutrition. In: L. I. Chiba, editor, Sustainable Swine Nutrition. John Wiley & Sons, Inc., Hoboken, NJ, USA. p. 3–22.

[CIT0027] Pearce, S. C., N. K. Gabler, J. W. Ross, J. Escobar, J. F. Patience, R. P. Rhoads, and L. H. Baumgard. 2013. The effects of heat stress and plane of nutrition on metabolism in growing pigs. J. Anim. Sci. 91:2108–18. doi: 10.2527/jas.2012-5738 23463563

[CIT0028] Pedersen, B. K., and T. N. Madsen. 2001. Monitoring water intake in pigs: prediction of disease and stressors. In: Livest. Env. VI: Proc. 6th Int. Symp. (21–23 May, Louisville, Kentucky, USA). Editors: R. R. Stowell, R. Bucklin, and R. W. Bottcher. ASAE Pub #701P0201.

[CIT0029] Petherick, J. C. 1983. A biological basis for the design of space in livestock housing. In: S. H. Baxter, M. R. Baxter, and J. A. D. MacCormack, editors, Farm Animal Housing and Welfare. Martinus Nijoff Publisher, Boston, MA, USA. p. 103–120.

[CIT0030] Quiniou, N., P. Massabie, and R. Granier. 2000. Diurnally variation of ambient temperature around 24 or 28 °C: Influence on performance and feeding behavior of growing pigs. In Proceedings of the First International Conference of Swine Housing, American Society of Agricultural Engineers, Des Moines, IA. p. 232–239. doi: 10.13031/2013.110.

[CIT0031] Renaudeau, D., J. L. Gourdine, and N. R. St-Pierre. 2011. A meta-analysis of the effects of high ambient temperature on growth performance of growing-finishing pigs. J. Anim. Sci. 89:2220–30. doi: 10.2527/jas.2010-3329 21297065

[CIT0032] Shaw, M. I., A. D. Beaulieu, and J. F. Patience. 2006. Effect of diet composition on water consumption in growing pigs. J. Anim. Sci. 84:3123–32. doi: 10.2527/jas.2005-690 17032808

[CIT0033] Sipos, W., S. Wiener, F. Entenfellner, and S. Sipos. 2013. Physiological changes of rectal temperature, pulse rate and respiratory rate of pigs at different ages including the critical peripartal period. Vet. Med. Austria. 100(3):93–98.

[CIT0034] Tavares, J. M. R., P. Belli Filho, A. Coldebella, and P. A. V. Oliveira. 2014. The water disappearance and manure production at commercial growing-finishing pig farms. Livest. Sci. 169:146–154. doi: 10.1016/j.livsci.2014.09.006.

[CIT0035] Turner, S. P., S. A. Edwards, and V. C. Bland. 1999. The influence of drinker allocation and group size on the drinking behaviour, welfare and production of growing pigs. Anim. Sci. 68:617–624. doi:10.1017/s1357729800050645

[CIT0036] Wallis, L. A., M. Healy, M. B. Undy, and I. Maconochie. 2005. Age related reference ranges for respiration rate and heart rate from 4 to 16 years. Arch. Dis. Child.. 90:1117–21. doi: 10.1136/adc.2004.068718 16049061 PMC1720181

[CIT0037] Xin, H., and J. A. DeShazer. 1991. Swine responses to constant and modified diurnal cyclic temperatures. Trans. ASABE. 34:2533–2540. doi: 10.13031/2013.31903.

